# Laryngeal inflammatory myofibroblastic tumor (IMT): a case report and review of the literature

**DOI:** 10.1186/s13256-016-0967-7

**Published:** 2016-06-23

**Authors:** Sok Yan Tay, Abhilash Balakrishnan

**Affiliations:** 1 Department of Otolaryngology Head and Neck Surgery, National University Health System, 5 Lower Kent Ridge Road, Singapore, 119074 Singapore; 2 Department of Otolaryngology, KK Women’s and Children’s Hospital, 100 Bukit Timah Road, Singapore, 229899 Singapore

**Keywords:** Laryngeal tumor, Hoarseness, Endoscopic, Benign, Recurrence

## Abstract

**Background:**

This case report is interesting as cases of children with laryngeal inflammatory myofibroblastic tumor are not common and previously had been presented as isolated case reports. This is the first case report in Asia describing a laryngeal inflammatory myofibroblastic tumor and its removal using an endoscopic approach.

**Case presentation:**

Our patient is a 12-year-old Malay girl from Singapore who presented with hoarseness without respiratory distress. The initial impression was that of a granuloma or a papilloma. We did a biopsy, which confirmed the histology to be inflammatory myofibroblastic tumor, and a magnetic resonance imaging scan showed a contrast-enhanced lesion. The lesion was excised completely using an endoscopic approach. The child was discharged well on the first postoperative day and she has been on follow-up for a year in the clinic.

**Conclusions:**

This report highlights the importance of understanding the differential diagnosis for a child with hoarseness. It is not uncommon for a pediatrician, a general practitioner, and a pediatric otolaryngologist to see a child presenting with hoarseness. In most cases, the diagnosis made would be screamer’s nodules, which is commonly seen in children. In a small group, recurrent respiratory papillomatosis form the diagnosis. Over the past few years, the cases of recurrent respiratory papillomatosis have decreased significantly. Laryngeal tumors are not common in children. However, we must maintain a high index of suspicion when we have a child with hoarseness who does not improve with speech therapy and watchful waiting. In such situations, a stroboscope is usually necessary to diagnose the voice problems and to rule out pathological conditions such as laryngeal tumors. If left untreated, the lesion can grow with time and result in a life-threatening airway condition. We also demonstrate our endoscopic technique in this report, and it has proven to be safe with no increased recurrence and much lower morbidity.

## Background

Inflammatory myofibroblastic tumors (IMT) are rare tumors that have a wide spectrum of histological features ranging from plasma cell-rich lesions to myofibroblast-predominant lesions. These lesions are frequently found in the lung but similar lesions have been reported in extrapulmonary sites such as the genitourinary tract, gastrointestinal tract, the breast, salivary glands, sinonasal tract, orbit, and the central nervous system. Laryngeal involvement of IMT is very rare. The largest case series of eight cases of laryngeal IMT was reported by Wenig *et al.* in 1995 [1].

IMT are rare benign tumors. They are also known as plasma cell granuloma. Although benign in nature, they tend to be locally aggressive and it is not uncommon for local invasion and recurrences to occur [2]. Complete excision has been the mainstay of treatment.

Laryngeal IMT has been reported in the pediatric population previously. However, they were mainly isolated cases. We present our unusual case of IMT in a 12-year-old Malay girl who presented to us with hoarseness with no airway issues.

## Case presentation

Our patient was a 12-year-old Malay girl from Singapore who presented to our clinic with the complaint of hoarseness for a duration of 9 months after a sore throat. Prior to that she was well. Unlike the typical presentation in recurrent respiratory papillomatosis, when the child tends to present earlier, at the age of 4 or 5 years old, her onset of hoarseness started only when she was 12 years old. It was progressively worsening. Fortunately, she did not have any associated airway issues. She was able to eat and drink normally and there was no suggestion of recent weight loss. The risk factors for hoarseness such as vocal abuse, talking, and singing loudly were also not present in her case. There was no significant family history of note.

A perceptual evaluation of voice quality using GRBAS (Grade, Roughness, Breathiness, Asthenia, and Strain) was performed. She was given a score of G: 3 R: 3 B: 0 A: 3 S: 1.

**Fig. 1:**
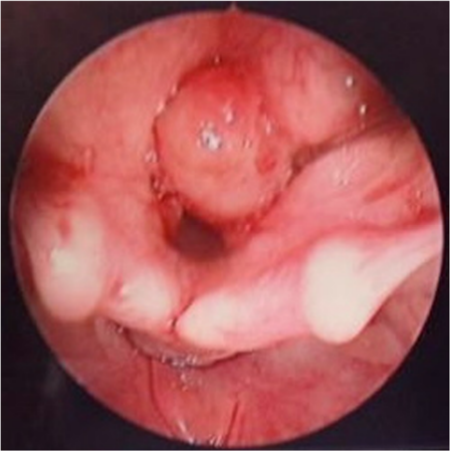
Pre-operatively, lesion on the right vocal cord as seen on flexible nasoendoscopy

Her only complaint was hoarseness. She was otherwise well. There were no signs of airway distress and no feeding issues. Her growth centile was appropriate for her age. There was no family history of similar disease. We performed a flexible nasoendoscopy on her. There was a lesion seen on her right vocal cord as shown in the picture (Fig. 1). This lesion was well circumscribed with a smooth mucosal surface. There were no other abnormalities. Her vocal cord movements were normal.

At that time, our working diagnosis for her included vocal cord polyp, granuloma, and recurrent respiratory papilloma.

**Fig. 2:**
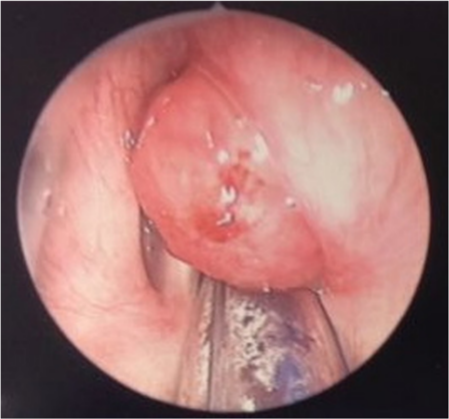
Intra-operatively, lesion seen on the anterior two thirds of the right true vocal cords and ventricle

**Fig. 3:**
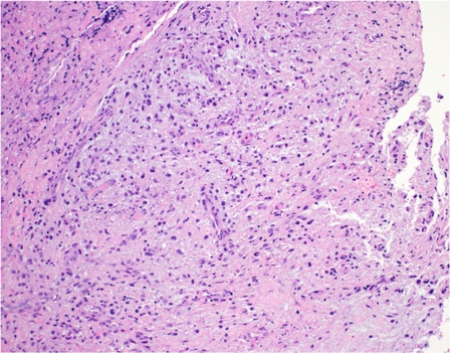
Myxoid tumour composed of ovoid to spindle shaped cells with associated plasma cells (H & E, magnification x 200)

**Fig. 4:**
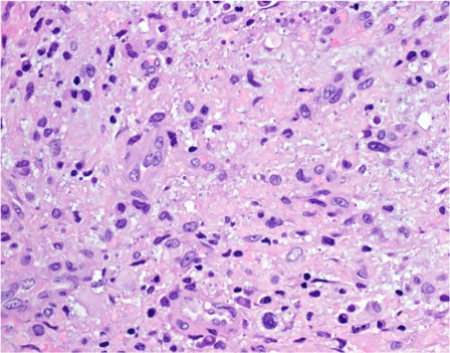
Cells demonstrating vesicular to hyperchromatic nuclei with ample eosinophilic to lightly basophilic cytoplasm (H & E, magnification x 400)

Our patient was brought to the operating theater where she underwent microlaryngoscopy and bronchoscopy (MLB). Intraoperatively, there was a large broad-based lesion involving the anterior two-thirds of her right true vocal cords and ventricle. The lesion was firm on palpation. Her left vocal cord was normal (Fig. 2). A biopsy was taken and sent for histology. Pathological analysis of the lesion revealed chronic inflammation with stromal myxoid degeneration and hyalinization (Figs. 3 and 4).

**Fig. 5:**
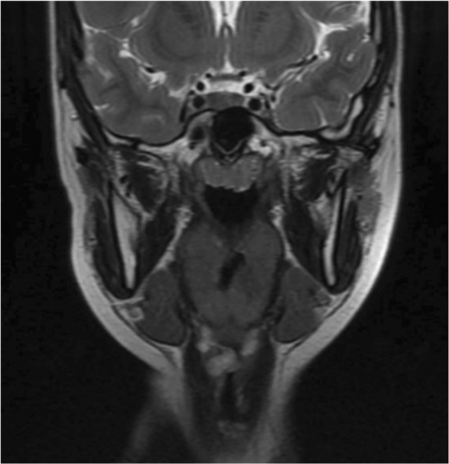
Coronal T2 image showing hyperintense lesion

**Fig. 6:**
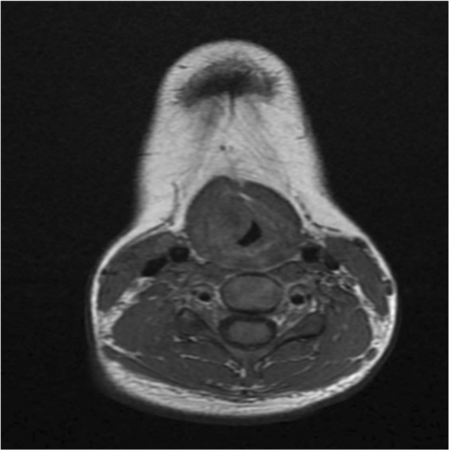
Axial T1 image showing isointense to hypointense lesion

**Fig. 7:**
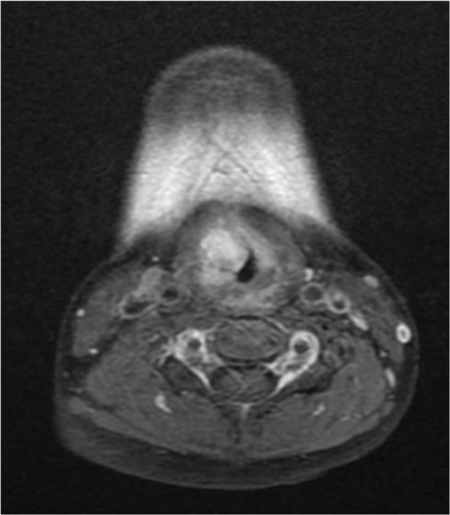
Axial T1 image with contrast showing lesion with avid enhancement

In view of the unusual location and presentation of the lesion, we decided to perform a magnetic resonance imaging (MRI) scan of her neck. The MRI scan showed a 1.5 × 1.6 × 1.7 cm heterogeneous submucosal solid lesion, isointense to hypointense on T1, and hyperintense on T2, with avid enhancement post contrast administration in the ventricular region on the right side (Figs. 5, 6 and 7).

**Fig. 8:**
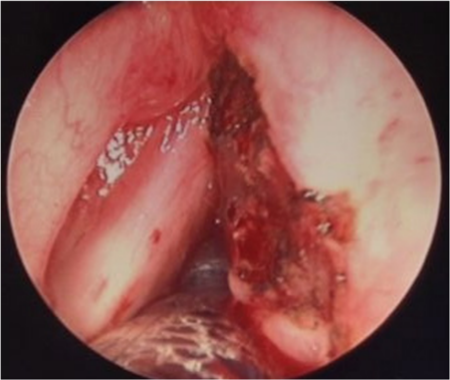
Post excision of the lesion

We proceeded to perform a complete excision of the lesion. We used the monopolar diathermy to define the excision margin. A laryngeal microdebrider was used to remove the lesion with endoscope assistance (Fig. 8). The child was monitored overnight in the hospital and was discharged home the following day.

The final pathologic analysis suggested the possible diagnosis of IMT.

Histopathology of the biopsy samples (Figs. 3 and 4) revealed myxoid changes and associated atypical-appearing cells characterized by ovoid to spindle-shaped cells with enlarged vesicular to hyperchromatic nuclei but still with an ample amount of eosinophilic to slightly basophilic-appearing cytoplasm, as well as associated multinucleated cells. These features were suggestive of an inflammatory myofibroblastic tumor (IMT).

Immunohistochemical staining showed these cells to have weak reactivity for smooth muscle actin and muscle-specific actin but negative for ALK1, desmin, and S100 protein. A low proliferation rate of less than 5 % was seen by Ki67 staining. CD68 was positive in scattered inflammatory cells but not the atypical cells.

**Fig. 9:**
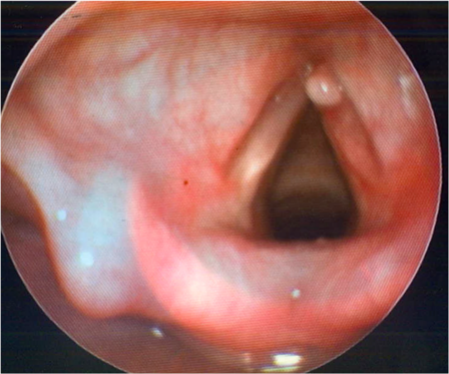
Nodule on the right vocal cord

**Fig. 10:**
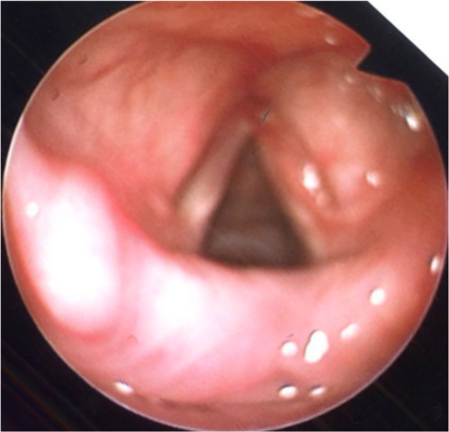
4 months later, previously seen nodule resolved

**Fig. 11:**
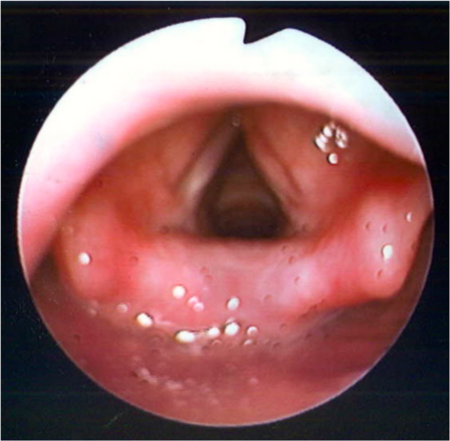
Last follow up picture showing no recurrence of the lesion

Following the excision, our patient was reviewed in the clinic. At 2.5 months post surgery, her voice has improved. There was a small nodule seen on her right vocal cord (Fig. 9). Our patient and her family were not keen to have repeat surgery for removal of the nodule in view of the symptom improvements. Four months later, she was reviewed again in the clinic. Her voice continued to improve and the previously seen nodule has disappeared (Fig. 10). We postulated that the nodule could have been a result of incorrect vocal usage post surgery and not related to the original IMT. She is currently on regular follow-up and will be returning 6 monthly for review. Her last review was about a year post surgery; there has been no recurrence of the lesion (Fig. 11).

## Discussion

Inflammatory myofibroblastic tumors (IMT) are benign lesions that mimic neoplastic lesions. To date, 39 cases of laryngeal IMT have been described in the English literature. The mean age of disease presentation was 44.5 years with a range of 2 to 74 years old [3]. Coffin *et al*. reported 84 extrapulmonary cases of IMT in a group of patients ranging from 3 months to 46 years and in this study only three out of the 84 patients had laryngeal IMT and they were all pediatric cases. The larynx is an uncommon site for IMT to occur [4].

IMT have been previously reported under a variety of names such as plasma cell granuloma, histiocytoma, plasma cell histiocytoma complex, inflammatory fibrosarcoma, and benign myofibroblastoma. However, since 1994, based on the World Health Organization (WHO) guidelines, the terminology inflammatory myofibroblastic tumor (IMT) has been used to describe these lesions [5]. IMT are characterized by myofibroblastic spindle cells on a background of inflammatory cells. Based on Coffin’s classification, it can be divided into three main categories: (1) hypocellular, (2) hypercellular, and (3) collagen sheets pattern. In this first type, the myofibroblasts are organized loosely within a background of inflammatory cells such as plasma cells, lymphocytes, and eosinophils. The second type are densely aggregated, elongated, short spindle cells or stellate cells arranged on a collagenized background, and in the third type, the cellular component is loosely arranged, resembling scar tissue [5–7]. Immunohistochemical staining often plays an important role in the diagnosis of laryngeal IMT. Muscle-specific actin, vimentin, and smooth muscle actin are often present in IMT. Occasionally, desmin and cytokeratins can be stained positive as well [8].

A child with IMT often presents with similar symptoms as patients with other laryngeal lesions. Hoarseness is the most common complaint. Other symptoms include that of dysphagia, failure to thrive, dyspnea, or stridor. The differential diagnosis to consider in a child who present with hoarseness would include that of recurrent respiratory papillomatosis, vocal cord polyp, and granulomatous lesion [9]. The appearance of IMT on endoscopy has been described as a smooth surface, polypoid or pedunculated lesion, firm and fleshy in nature. The size of which can range from several millimeters to 6 cm [1, 10].

The true causative factor leading to the development of laryngeal IMT remains unknown. Many theories have been proposed and these include trauma, infections, neoplasm, and immunological factors. Smoking has been suggested as a risk factor, as 22.6 % of the patients with laryngeal IMT were smokers. Epstein–Barr virus (EBV) has been shown to be associated with IMT at extralaryngeal sites but not in the larynx [8, 11]. The neoplastic theory has been suggested because of tumor recurrence and malignant progression seen in cases of IMT. Anaplastic lymphoma kinase 1 (ALK 1), chromosomal rearrangement at chromosome 2p23 and p80, have been suggested to be associated with the neoplastic theory [12].

Laryngeal IMT occurs most commonly in the true vocal cords, followed by the subglottis. Rarely, do they occur in the supraglottis [13]. The presentation complaints vary depending on the site of the lesion. In our case and similar to previous case series, voice change is the most common presentation (74 %), followed by stridor (29 %) and dyspnea, shortness of breath (22.5 %), and globus sensation (16 %).

Prior to surgery, radiological investigations such as computed tomography (CT) scans and magnetic resonance imaging (MRI) are usually performed to determine the extent of the lesion. An MLB with biopsy and immunohistochemical staining of the lesion is often necessary for diagnosis. Surgical excision with good margins is the treatment of choice for this disease. This can be done using an endoscopic technique or an open technique. Endoscopic excision using laser or cold steel instruments is the preferred technique. Open technique is commonly reserved for recurrent cases, cases with poor visualization using endoscope or when malignancy cannot be excluded. We used the endoscopic technique in this case and were able to excise the lesion completely. The advantages of adopting the endoscopic technique were lesser postoperative pain, faster recovery, shorter hospital stay, and better voice outcome. The exposure from the endoscope was adequate for us to visualize the entire lesion and to perform a complete excision. However, as this is our first case, we are still following up this patient closely. At 1 year post surgery, our patient is still disease-free without any recurrence.

Radiotherapy is used mainly to treat local recurrence. The use of chemotherapy is also limited to the treatment of lesion that has undergone malignant transformation or recurrent IMT. Steroid has been used on its own and also as an adjunct with surgery. Steroid as a sole therapy seems to be less effective. However, in a patient with severe comorbidity and surgery carries a high risk, or when the surgery will result in significant function loss, a course of pulsed steroids can be attempted.

The main factors leading to recurrence of disease is often partial/incomplete excision of tumor. The recurrence rate of laryngeal IMT is 8 to 18 % [9, 14]. The recurrence occurs most commonly within 2 to 12 months. In some patients with multiple recurrences, a laryngectomy may have to be considered. We are pleased that our patient has been disease-free throughout the follow-up at the time of submission.

## Conclusions

Inflammatory myofibroblastic tumor is an unusual tumor that can mimic a neoplastic process. Many etiologies have been proposed but none has been proven. Immunohistochemical staining plays an important role in the histological diagnosis of IMT. The initial presentation of IMT in a child is most often hoarseness. It is important to exclude laryngeal lesion as a cause of persistent hoarseness in a child. This can be done with the use of a nasoendoscopy. A delay in diagnosis of a laryngeal lesion can result in a life-threatening airway issue. The treatment of choice for IMT remains surgical excision with good margins. The endoscopic approach used in this case results in less postoperative morbidity with no increased recurrence. Good postoperative follow-up is necessary in order to detect any recurrence promptly.

## Abbreviations

CT, computed tomography; IMT, inflammatory myofibroblastic tumor; MLB, microlaryngobronchoscopy; MRI, magnetic resonance imaging
